# DSMUNet: A Lightweight Model for Road Crack Segmentation

**DOI:** 10.3390/s26103055

**Published:** 2026-05-12

**Authors:** Yunqing Liu, Xu Du, Chunting Zuo

**Affiliations:** 1 School of Electronic Information and Engineering, Changchun University of Science of Technology, Changchun 130118, China; mzlyq@cust.edu.cn (Y.L.); 2023200138@mails.cust.edu.cn (X.D.); 2 School of Electronic Information and Engineering, Changchun Guanghua College, Changchun 130118, China

**Keywords:** road maintenance, image segmentation, lightweight model, deep learning

## Abstract

As the most common safety hazard among road surface distresses, pavement cracks require fine-grained segmentation to support subsequent maintenance decision-making. However, existing methods find it difficult to cope with complex environmental interference and multi-morphology cracks while maintaining low computational cost. To address the above issues, this study proposes a lightweight crack segmentation model, DSMUNet. Based on the U-shaped encoder–decoder framework, depthwise separable convolution is adopted to replace traditional convolution to reduce computational cost, and the SGE spatial group enhancement mechanism is introduced at the encoder side to emphasize crack features and suppress interference from non-crack textures. In addition, a new multi-scale feature fusion module is proposed to improve the overall connectivity of cracks during the feature fusion stage, thereby further improving model performance on the basis of model lightweighting. The experimental results show that DSMUNet contains only 0.55 M parameters and requires 21.708 GFLOPs, with an average inference latency of 5.42 ms, while achieving Dice/mIoU scores of 78.10%/69.05% on the private dataset and 87.12%/79.57% on the Crack500 dataset, respectively. These results demonstrate the advantages of this study in terms of resource consumption as well as its excellent segmentation performance, providing a more efficient implementation scheme for pavement crack segmentation in resource-constrained environments.

## 1. Introduction

Pavement cracks are the most common external feature of structural pavement distress [1,2]. If small cracks at the early stage are not detected and treated in time, they can easily expand rapidly under the combined effects of vehicle loads, natural wear, and other factors, thereby further causing more severe pavement damage, significantly shortening the service life of the road, increasing maintenance costs, and creating safety hazards [3,4,5].

Traditional pavement inspection requires engineers to make on-site judgments on whether damage exists in a certain area and to determine the damage type and severity based on experience. This kind of manual inspection technique has disadvantages such as heavy workload, low efficiency, and strong subjectivity [6,7,8]. In recent years, the continuous improvement of image acquisition equipment and the rapid development of digital image technology have also introduced new ideas for crack detection [9]. During this period, pavement crack detection mainly relied on traditional image processing methods, such as threshold segmentation [10,11] and edge segmentation [12].

In threshold-based approaches, pixels are classified as crack or non-crack according to grayscale intensity; although these methods are simple and computationally efficient, they are highly sensitive to illumination changes and shadows, leading to frequent misclassification and missed detections [13]. By contrast, edge-based methods emphasize crack-boundary features; however, substantial interference can be introduced by noise and background textures, and edges are prone to fragmentation. Consequently, extensive post-processing is often required, which limits their practical applicability.

With the development of deep learning techniques, an increasing number of studies have applied deep neural networks to pavement crack detection and segmentation tasks. Existing methods can generally be categorized into encoder–decoder-based methods, multi-scale contextual modeling methods, and attention-enhanced methods.

Among them, encoder–decoder-based methods extract semantic features through the encoder, recover spatial resolution through the decoder, and supplement low-level detailed information via skip connections. Therefore, they have been widely used for pixel-wise pavement crack segmentation. For example, RUC-Net [14] combines U-Net with ResNet to improve pixel-level pavement crack segmentation performance, while EDNet [15] models the imbalance between crack pixels and background pixels. Such methods can effectively preserve crack boundaries and spatial details. However, standard convolutions and multi-layer feature stacking also introduce relatively high numbers of parameters and computational costs.

Multi-scale contextual modeling methods mainly enlarge the receptive field through dilated convolution, pyramid pooling, or multi-scale feature aggregation, thereby adapting to crack targets with different widths and morphologies. For example, CrackSegNet [16] combines dilated convolution, spatial pyramid pooling, and skip connections to enhance the multi-scale feature representation of crack regions. Some pyramid-structured crack detection networks employ dilated convolutions to reduce the loss of detailed information caused by downsampling. Pavement distress segmentation methods based on DeepLabv3+ [17] also enhance multi-scale semantic modeling capability through structures such as ASPP. These methods can improve the recognition of cracks at different scales, but multi-branch contextual structures usually introduce additional computational overhead.

Attention-enhanced methods mainly highlight crack-related responses and suppress interference from complex backgrounds through spatial attention, channel attention, and related mechanisms. For instance, ADDU-Net [18] uses an asymmetric dual-decoder structure and dual-attention modules to capture the features of both coarse and fine cracks. Context-CrackNet [19] improves the segmentation of tiny cracks through region-focused enhancement and context-aware modules. Such methods can enhance the discriminative ability of models for crack regions. Nevertheless, the placement and computational cost of attention structures still affect their applicability in lightweight scenarios.

In recent years, lightweight pavement crack segmentation methods have gradually attracted increasing attention. Related studies mainly reduce model complexity by employing depthwise separable convolutions, lightweight attention mechanisms, or hybrid lightweight architectures. For example, EGA-UNet [20] introduces efficient lightweight convolutional modules to address road cracks under complex backgrounds and varying scales. LPCD-Net [21] reduces the computational burden of pavement crack detection from the perspective of a lightweight encoder–decoder architecture. S2TNet [22] integrates lightweight crack segmentation with crack tracking on robotic platforms, further demonstrating the practical demand for this research direction.

Although the above methods have achieved certain progress, pavement crack segmentation in real-world environments still faces many challenges. First, pavement backgrounds contain numerous interference factors with appearances similar to cracks, such as asphalt textures, repair traces, lane markings, stains, and shadow boundaries. These structures can easily produce misleading responses in the feature space, thereby causing false detections and missed detections. Second, pavement cracks are complex in type and exhibit significant morphological variations. Various distress patterns, including linear cracks, mesh cracks, block cracks, and cracks near repaired regions, may coexist in the same scene. Different types of cracks differ markedly in width, propagation direction, branching structure, connectivity, and texture contrast with the background. These differences lead to problems such as blurred crack boundaries, discontinuous local structures, and incomplete recognition of fine cracks. Third, many high-performance segmentation networks usually improve segmentation accuracy by increasing network depth, expanding channel width, or introducing complex structures. This significantly increases the number of model parameters and computational cost, thereby limiting their application in mobile, vehicle-mounted, and embedded pavement inspection scenarios.

To address the problem of balancing complex pavement background interference, multi-type crack morphologies, and lightweight deployment constraints, this paper constructs a lightweight pavement crack segmentation network named DSMUNet. The proposed model is designed according to the characteristics of crack segmentation tasks and jointly considers lightweight feature extraction, interference suppression at the encoder side, and multi-scale fusion at the decoder side. Depthwise separable convolutions are adopted to reduce the number of parameters and computational complexity of the backbone network. A spatial group enhancement mechanism is introduced into the encoder so that crack-related features can be strengthened before entering the skip connections, while crack-like background interference is suppressed. After the concatenation of skip-connection features in the decoder, a gated multi-scale feature fusion module is designed to supplement crack structural information under different receptive fields, thereby enhancing the continuous representation of slender cracks, branching cracks, and locally discontinuous cracks.

The main contributions of this study are as follows:

1. This study collects and constructs a multi-morphology asphalt pavement crack dataset containing various environmental interference factors, including strong illumination, shadows, occlusions, and repaired pavement surfaces. The dataset consists of 3659 high-resolution images with pixel-level mask annotations, providing a more challenging and representative experimental basis for subsequent research.

2. This study designs a gated multi-scale feature fusion module for the decoder fusion stage. The module supplements crack structural information under different receptive fields through multi-scale depthwise separable dilated convolution branches, and employs mean aggregation, group normalization, and a residual gating mechanism to control additional computational overhead and feature disturbance.

3. This study proposes a lightweight pavement crack segmentation network named DSMUNet. Based on an encoder–decoder architecture, the network adopts depthwise separable convolutions to reduce the number of parameters and computational complexity. A spatial group enhancement mechanism is introduced in the encoder to suppress crack-like background interference, while a gated multi-scale feature fusion module is introduced in the decoder to enhance multi-scale crack structure representation.

## 2. Methodology

### 2.1. DSMUNet

Pavement cracks are typically characterized by slenderness, irregularity, large scale variations, and local discontinuities. Meanwhile, structures such as asphalt textures, shadow boundaries, and repair traces in complex pavement backgrounds can easily produce responses similar to crack regions, posing considerable challenges to accurate segmentation. To address these issues, this paper proposes a lightweight pavement crack segmentation network, named DSMUNet, aiming to improve the feature representation capability and structural continuity of crack regions in complex scenarios while reducing the number of model parameters and computational complexity.

**Figure 1:**
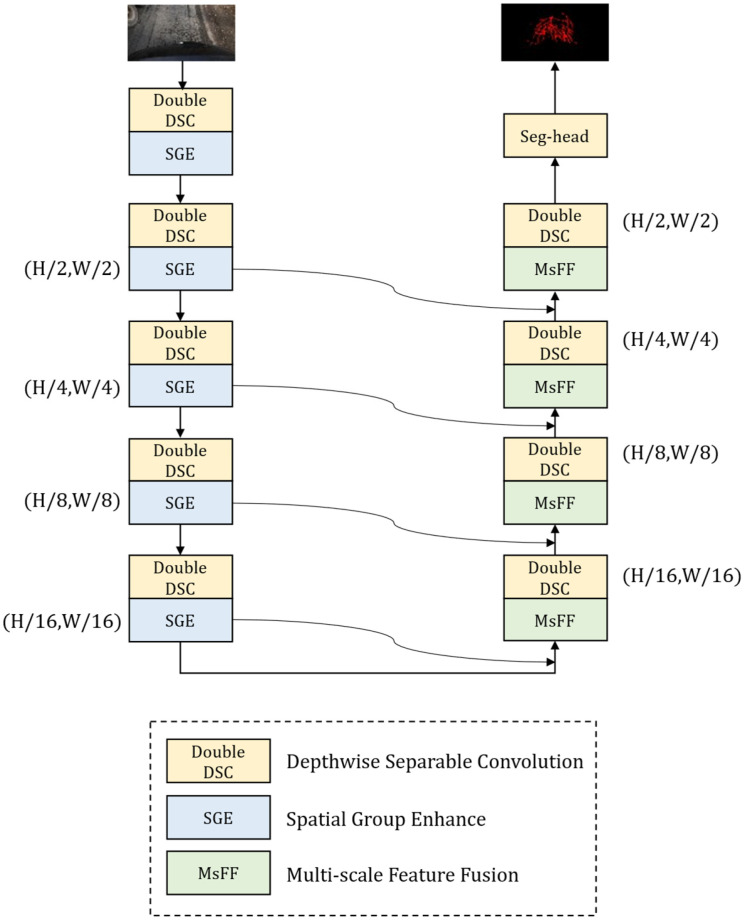
Overall Architecture of DSMUNet.

DSMUNet adopts an encoder–decoder architecture as its overall framework, as shown in Figure 1. To adapt to pavement crack segmentation tasks under resource-constrained conditions, depthwise separable convolutions are employed to replace standard convolutions in constructing the backbone feature extraction modules, thereby effectively reducing the number of model parameters and computational complexity.

In the encoding stage, to enhance the responses of crack-related regions and suppress crack-like background interference, a spatial group enhancement module is introduced after feature extraction at each scale. The features are recalibrated by this module before being transmitted to the decoder through skip connections. In the decoding stage, to improve the representation of crack structures at different scales, a gated multi-scale feature fusion module is designed after the concatenation of skip-connection features. This module supplements crack structural information under different receptive fields through multiple depthwise separable dilated convolution branches and combines a gating mechanism to achieve adaptive feature fusion, thereby enhancing the continuity of crack prediction results.

Compared with commonly used lightweight segmentation models, the design of DSMUNet is not limited to model compression. Instead, it is specifically configured for background interference suppression and structural continuity recovery in pavement crack segmentation. General lightweight models usually focus on reducing the number of parameters and computational complexity, while lacking targeted processing for crack-like interference such as asphalt textures, shadow boundaries, and repair traces. Lightweight U-shaped networks such as DCSAU-Net mainly emphasize compact feature representation, but pay relatively insufficient attention to the combination of interference suppression in the encoder and multi-scale structural compensation in the decoder.

The design of DSMUNet focuses on task-oriented configuration of different network stages for crack segmentation. Specifically, depthwise separable convolutions are used to construct a lightweight backbone, the spatial group enhancement module is used for feature enhancement in the encoder, and the gated multi-scale feature fusion module is used for structural compensation in the decoder. Through the above collaborative design, DSMUNet effectively improves crack segmentation performance under complex background conditions while maintaining low model complexity.

### 2.2. Lightweight Convolution Module

To achieve a lightweight model while preserving the ability to extract fine crack boundaries and details, U-Net was adopted as the baseline and its architecture was refined. By leveraging an encoder–decoder structure with skip connections, U-Net can partially alleviate the loss of spatial information caused by repeated downsampling. However, feature extraction and fusion in the original U-Net are typically implemented by stacking multiple standard convolutions. Because standard convolutions perform computations jointly across spatial and channel dimensions, both the parameter count and computational cost scale with the number of channels. As a result, when such operations are accumulated across different scale levels, a large parameter size and high inference overhead are often produced, directly affecting the model’s real-time performance and usability.

Meanwhile, pavement cracks are characterized by slender shapes, diverse morphologies, and irregular edges, and crack information often exists in the form of fine-grained features. During the successive downsampling and convolutional encoding in the encoder, these features are prone to being overwhelmed by interference from background patterns, resulting in insufficient crack connectivity, local fragmentation, and missed detections. If accuracy improvement is pursued solely by deepening the network or expanding channels, parameter redundancy and computational burden will be further amplified, and the lightweight objective cannot be satisfied.

**Figure 2:**
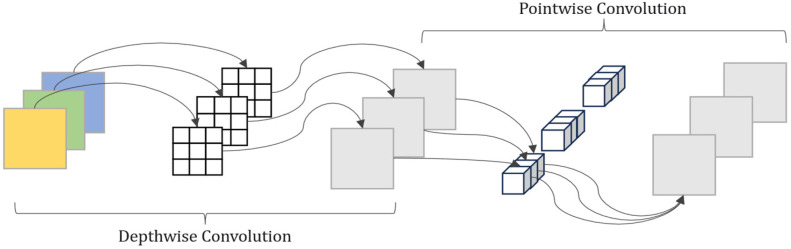
Depthwise Separable Convolution.

To address these requirements, depthwise separable convolution (DSConv) was introduced to replace part of the standard convolutions, thereby reducing the parameter count while maintaining effective representation learning, as illustrated in Figure 2.

DSConv consists of a depthwise convolution followed by a pointwise convolution (PwConv). Let the input feature map be $y\in R^{H\times W\times C_{\mathrm{in}}}$. In DwConv, convolution is performed independently for each input channel, and the output z is computed as shown in Equation (1):

(1) $$\begin{array}{cccc} & z_{i,j,c}=\sum_{u=0}^{K-1}\sum_{v=0}^{K-1}\delta_{DWconv}\left(u,v,c\right)\cdot y_{i+u,j+v,c} & & \end{array}$$

where $\delta_{\mathrm{DW}}$ denotes the depthwise kernel weights, $\left(i,j\right)$ indexes the spatial location, $\left(u,v\right)$ represents the kernel offset, and $c\in \{1,\ldots ,C_{\mathrm{in}}\}$ is the channel index.

Subsequently, PwConv applies a 1×1  convolution to fuse and project z along the channel dimension, producing an output feature map $x\in R^{H\times W\times C_{\mathrm{out}}}$. This operation is formulated as shown in Equation (2):

(2) $$x_{i,j,m}=\sum_{c=1}^{C_{in}}\delta_{PWconv}\left(c,m\right)\cdot z_{i,j,c}$$

where $\delta_{\mathrm{PW}}$ denotes the pointwise kernel weights and $m\in \{1,\ldots ,C_{\mathrm{out}}\}$ is the output channel index. By combining *DwConv* and *PwConv*, the overall *DSConv* process can be expressed as shown in Equation (3):

(3) $$DSConv\left(\delta_{DWconv},\delta_{PWconv},y\right)=PWConv\left(\delta_{PWconv},DWConv\left(\delta_{DWconv},y\right)\right)$$

From a parameter-efficiency perspective, for a kernel size of K×K with $C_{\mathrm{in}}$ input channels and $C_{\mathrm{out}}$ output channels, a standard convolution requires $K^{2}C_{\mathrm{in}}C_{\mathrm{out}}$ parameters, whereas DSConv requires only $K^{2}C_{\mathrm{in}}+C_{\mathrm{in}}C_{\mathrm{out}}$, thereby substantially reducing model size and inference cost.

In many existing works, *DSConv* is applied as a single *DSConv* layer. In this study, a design more consistent with U-Net conventions was adopted: the original DoubleConv block (two stacked 3×3 standard convolutions) was retained in form, but each 3×3 standard convolution was replaced with a *DSConv*. By cascading two *DSConv* layers, the parameter budget was reduced while feature extraction capability was preserved as much as possible, thereby better balancing segmentation accuracy and lightweight deployment requirements for pavement crack segmentation.

### 2.3. Spatial Group Enhancement

**Figure 3:**
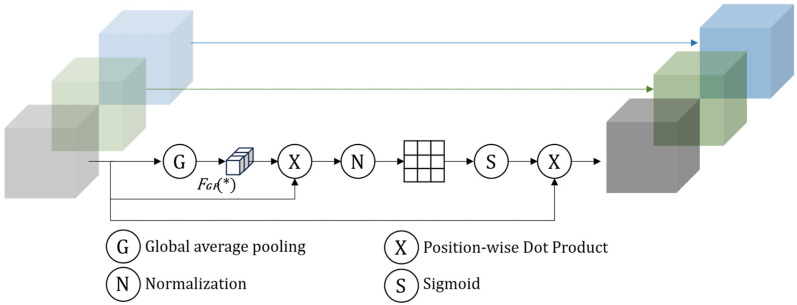
Spatial Group Enhancement module structure diagram.

Segmentation errors in pavement crack detection are rarely caused by cracks being completely invisible; instead, they predominantly arise from non-crack textures that exhibit crack-like appearances. When background patterns—such as asphalt aggregate textures, repair joints, or shadow boundaries—produce salient spurious activations in the feature space, the skip connections in conventional U-Net tend to pass these fine-grained but misleading details directly to the fusion stage. As a result, crack boundaries can be corrupted by background textures, leading to false annotations and local discontinuities (e.g., broken crack segments). To suppress such texture interference at an early stage and improve feature discriminability, a Spatial Group Enhancement (SGE) [23] module is introduced at each encoder layer. With only minor additional overhead, SGE highlights informative regions while attenuating irrelevant responses, thereby improving segmentation performance, as illustrated in Figure 3.

To further control computational cost, SGE is inserted only in the encoder. Let the input feature tensor be $x\in R^{B\times C\times H\times W}$, where B  denotes the batch size, C  is the number of channels, and H and W are the spatial height and width, respectively. In SGE, the channels are divided into G groups and rearranged by folding the group dimension into the batch dimension, yielding $x_{g}$ as shown in Equation (4):

(4) $$x\to x_{g}\in R^{\left(B\cdot G\right)\times \left(\frac{C}{G}\right)\times H\times W}$$

Next, a channel-wise global response is obtained within each group via global average pooling, followed by feature recalibration. On this basis, summation along the channel dimension produces a single-channel spatial map s. To reduce scale variation across samples and groups, s is normalized over the H×W spatial domain, and learnable group-level affine parameters $\left(w,b\right)\;$ are introduced. The resulting attention map a is obtained through a sigmoid activation, and the final output is produced via element-wise modulation, as shown in Equation (5):

(5) $$\left\{\begin{array}{c} a=\sigma \left(w\cdot Norm\left(s\right)+b\right)\\ y=x⊙a \end{array}\right.$$

### 2.4. Multi-Scale Feature Fusion Module

Pavement cracks commonly appear as irregular structures, including linear fissures and network-like alligator cracking, and multiple scales may coexist within the same image. In typical segmentation networks, the decoder is responsible for detail recovery and semantic alignment. However, when skip features are fused using convolution alone, it is often difficult to simultaneously preserve cross-scale continuity and maintain consistent crack width, which can result in discontinuous predictions, jagged boundaries, and unstable width estimation.

**Figure 4:**
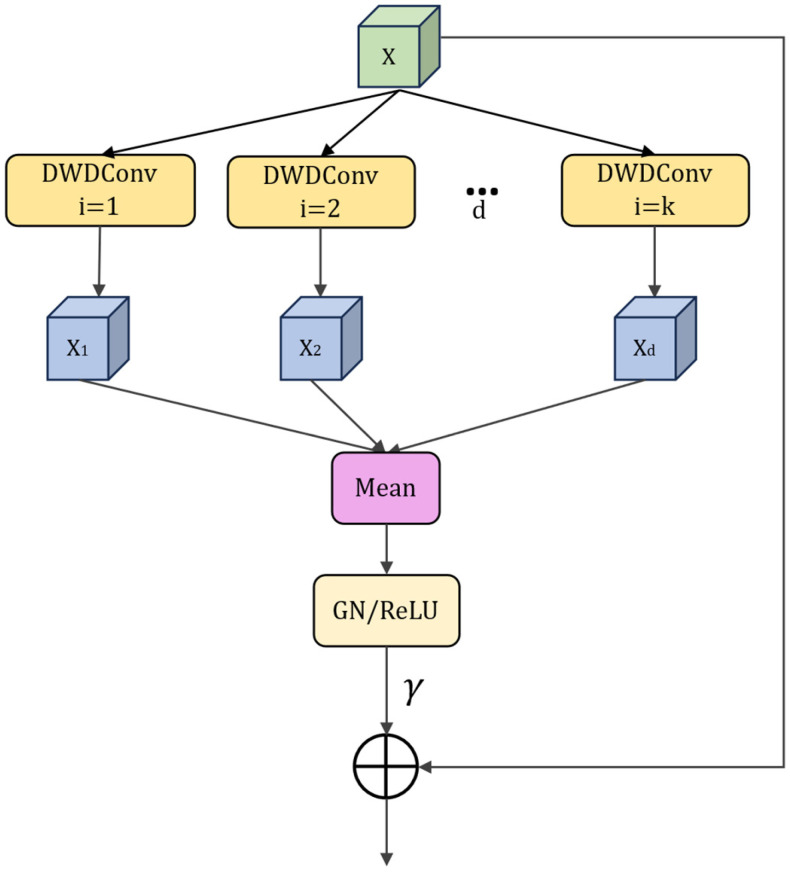
Multi-Scale Feature Fusion Module schematic.

To address these problems, a multi-scale feature fusion module was designed in this study. It was introduced after the skip-connection concatenation at each decoder level, and multi-scale processing was performed immediately after concatenation, thereby enhancing the adaptability of the decoded features to multi-scale structures. The structure is shown in Figure 4.

Specifically, the proposed module employs multi-branch depthwise dilated convolutions to process features in parallel under different receptive fields. A set of dilation rates from small to large is used to form multi-scale branches: branches with smaller dilation focus on local edges and fine-grained texture variations; branches with moderate dilation model crack directionality and local connectivity; and branches with larger dilation further expand the receptive field to capture longer-range structures, thereby better characterizing the global morphology of multi-form cracks. Let the dilation rate of the i-th branch be $d_{i}$. The branch output is computed as shown in Equation (6):

(6) $$\begin{array}{cccc} & f_{i}\left(x\right)=DWConv_{d_{i}}\left(x\right),i=1,2,\ldots ,K. & & \end{array}$$

For multi-branch fusion, a mean aggregation strategy is adopted to avoid channel expansion introduced by concatenation and to further control computational overhead. Given K branches, the fused feature is obtained as shown in Equation (7):

(7) $$\begin{array}{cccc} & f\left(x\right)=\frac{1}{K}\sum_{i=1}^{K}f_{i}\left(x\right). & & \end{array}$$

The fused features are then fed into normalization and activation layers. Because crack segmentation training is often limited by GPU memory, small batch sizes are commonly used; under such settings, Batch Normalization can suffer from unstable batch statistics and may impair training stability. To reduce reliance on batch-level statistics, Group Normalization is employed within this module in place of BatchNorm. GN normalizes features by grouping channels within each individual sample, making it independent of batch size and less sensitive to batch-size variations, which improves training stability and robustness.

Finally, the module produces its output in a residual manner and introduces a learnable gating factor γ, as shown in Equation (8):

(8) $$\begin{array}{cccc} & y=x+\gamma \cdot ReLU\left(GN\left(f\left(x\right)\right)\right). & & \end{array}$$

where *γ* denotes a learnable scalar gating parameter used to control the contribution of the multi-scale enhancement branch to the backbone features. In this study, *γ* is initialized to 0, so that the multi-scale branch does not introduce excessive perturbations to the feature distribution at the early stage of training. As training proceeds, *γ* is jointly optimized with the other network parameters, allowing the model to adaptively adjust the contribution of the multi-scale branch at different decoding stages.

To further verify whether the gated multi-scale branch is effectively utilized after training, the learned *γ* values were recorded over three independent runs with different random seeds. The results are shown in Table 1. It can be observed that the learned *γ* values are non-zero in most decoder stages after convergence, indicating that the multi-scale enhancement branch is not ignored by the network, but is incorporated into the decoding process. In addition, the *γ* values differ across different decoder stages, suggesting that different decoding layers have different degrees of dependence on multi-scale structural supplementation. The negative mean value observed at Up stage 3 further indicates that the gating factor does not simply amplify the multi-scale branch, but adaptively regulates its contribution according to the feature distribution at the corresponding stage.

This residual gating design is suitable for pavement crack segmentation tasks. Since the responses of fine cracks are usually weak, directly introducing a strong multi-scale enhancement branch at the early training stage may amplify crack-like background responses, such as asphalt textures, shadow boundaries, and repair traces, thereby affecting the convergence stability of the model. By initializing *γ* to 0, the network can first learn fundamental crack-discriminative features through the backbone path, and then gradually incorporate multi-scale structural information during subsequent optimization. In this way, the module provides adaptive structural supplementation for slender cracks, branching cracks, and locally discontinuous cracks at the feature fusion stage, helping to alleviate problems such as crack breakpoints, ambiguous bifurcations, and unstable crack widths.

### 2.5. Data Source

This study constructs a private asphalt pavement crack dataset. The images were collected by the research team from road scenes in Changchun, Jilin Province, China. The acquisition device was a high-resolution camera mounted on a vehicle platform. During data collection, the vehicle maintained a low speed of approximately 20–30 km/h to reduce the influence of motion blur on image quality. The camera was installed at the rear of the vehicle, with the lens facing the pavement surface. The mounting height was approximately 2.3 m above the ground, and the imaging angle was vertically downward. The detailed acquisition parameters are listed in Table 2.

**Figure 5:**
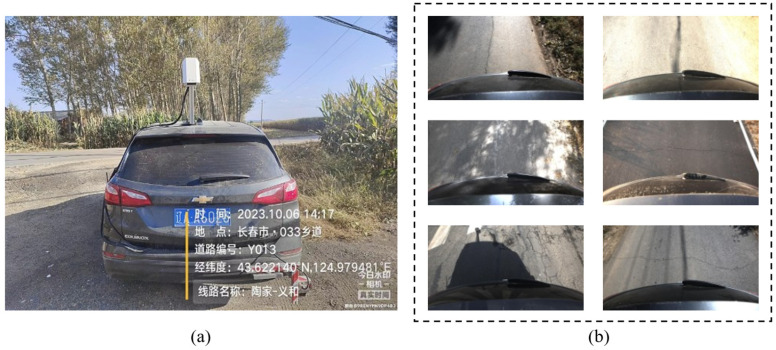
Dataset overview: (**a**) acquisition equipment used for data collection; (**b**) representative images of the constructed dataset.

To improve the representativeness of the dataset in complex road environments, various real-world interference factors were retained during data collection, including strong illumination, shadows, non-uniform lighting, pavement repair traces, asphalt textures, and local occlusions. Meanwhile, the dataset contains multiple crack morphologies, such as linear cracks, mesh cracks, block cracks, and cracks near repaired regions, as shown in Figure 5. After preliminary screening and quality control, a total of 3659 representative crack images were obtained for subsequent model training and evaluation.

Subsequently, three domain experts used the LabelMe annotation tool to generate pixel-level ground-truth masks. The annotation categories included two classes: crack regions and background regions. Specifically, crack pixels were labeled as the foreground class, while background pixels were labeled as the background class. To ensure the consistency and reproducibility of the annotation results, a unified pixel-level annotation protocol was established in this study. Crack pixels were defined as the visible body regions of pavement cracks. The annotation boundaries were required to fit the actual crack contours as closely as possible, while avoiding artificial expansion. For complex cases such as shadows, reflections, and stains, only the visible crack regions in the image were annotated, and completely invisible regions were not inferred or completed manually. Non-crack structures, such as road markings, regular joints, cutting seams, and strong reflective strips, were treated as background.

In terms of annotation quality control, a small set of difficult samples containing shadows, crack intersections, and background interference was first selected for trial annotation. Based on this process, the annotation criteria were unified and a rule checklist was formulated. During formal annotation, annotator self-checking and difficult-sample marking mechanisms were implemented, and each batch of data was randomly inspected. For samples with controversial annotations or unclear boundaries, the three experts conducted a joint discussion, and, when necessary, a senior annotator made the final decision to determine the final mask. After standardization, all annotation results were exported in PNG format at a resolution of approximately 2048 × 1080 pixels for subsequent model training, validation, and testing.

In addition, to further verify the generalization ability of the proposed model under different acquisition devices, scene conditions, and data distributions, the public Crack500 dataset [24] was introduced as supplementary experimental data. The Crack500 dataset contains 500 road crack images captured by smartphones and exhibits strong scene diversity and practical representativeness. Compared with the private dataset constructed in this study, Crack500 differs in terms of imaging device, shooting perspective, and pavement texture characteristics. Therefore, it can be used to more comprehensively evaluate the recognition and segmentation performance of the model for different types of road cracks, thereby improving the objectivity and persuasiveness of the experimental results.

## 3. Results

### 3.1. Experimental Setup

All experiments in this study were conducted on a high-performance desktop workstation running the Windows operating system (self-assembled, Changchun, China). The experimental device was equipped with an NVIDIA GeForce RTX 5090D GPU (Santa Clara, CA, USA) with 32 GB of memory. All methods were implemented and evaluated in a Python 3.12 environment. To ensure the controllability and reproducibility of the experimental comparisons, a unified training protocol was adopted for all comparative and ablation experiments. Specifically, the dataset was divided into training, validation, and test sets at a ratio of 8:1:1. All input images were uniformly resized to 640 × 640 before being fed into the models. The maximum number of training epochs was set to $E_{max}=200$, the batch size was set to B=2, and the initial learning rate was set to $1\times 10^{-2}$. The model parameters were optimized using the Adam optimizer.

The purpose of adopting a unified training protocol was to control differences in training conditions, enabling different models to be compared under the same data split, input resolution, and training budget, thereby reducing the influence of additional training factors on the experimental results. Meanwhile, this setting helped avoid subjective bias caused by different degrees of model-specific hyperparameter tuning, making the comparison process more consistent. For the ablation experiments, the unified training protocol also reduced the interference of training strategy variations in analyzing the contributions of different modules, making the effects of individual components more interpretable. To improve the stability and reliability of the experimental results, each comparative model was independently trained three times with different random seeds. The model weights that achieved the best performance on the validation set were then used for testing to ensure the reliability of the final results.

### 3.2. Evaluation Metrics

In this study, Recall, mean Intersection over Union (mIoU), and F1-score are adopted as the primary metrics for evaluating segmentation performance. Let K denote the number of classes (crack/background in this work, thus K=2). Pixel-level true positives $\left(TP\right)$, false positives $\left(FP\right)$, and false negatives $\left(FN\right)$ are defined in the standard manner for each class k.

mIoU is a widely used semantic-segmentation metric that quantifies the overlap between the predicted mask and the ground-truth mask. It is computed as the mean IoU over all classes, as shown in Equation (9):

(9) $${mIoU}_{k}=\frac{1}{K}\sum_{k=1}^{K}\;\frac{TP_{k}}{TP_{k}+FP_{k}+FN_{k}}$$

Recall measures the proportion of ground-truth positive pixels that are correctly identified, reflecting the model’s ability to reduce missed detections. It is defined in Equation (10):

(10) $${Recall}_{k}=\frac{TP_{k}}{TP_{k}+FN_{k}}$$

The Dice evaluates the overlap between the predicted and ground-truth regions by computing twice the intersection divided by the sum of pixel counts, as shown in Equation (11):

(11) $$\begin{array}{cccc} & {Dice}_{k}=\frac{2TP_{k}}{2TP_{k}+FP_{k}+FN_{k}} & & \end{array}$$

The F1-score is the harmonic mean of Recall, providing a single measure that balances false alarms and missed detections, as given in Equation (12):

(12) $$F1_{k}=\frac{2TP_{k}}{2TP_{k}+FP_{k}+FN_{k}}$$

In addition, to assess model efficiency and deployment friendliness, the number of parameters (Params) and floating-point operations (GFLOPs) are reported. Params reflect the storage cost and structural complexity of the model and are defined as the total number of learnable parameters, as shown in Equation (13):

(13) $$Params=\sum_{l=1}^{L}N_{l}$$

where L denotes the number of network layers and $N_{l}$ represents the number of learnable parameters in the l-th layer. In general, smaller Params indicate lower memory/storage requirements and better suitability for edge deployment.

GFLOPs measure the theoretical computational cost of a single forward pass (in billions of floating-point operations), as defined in Equation (14):

(14) $$GFLOPs=\frac{FLOPs}{10^{9}}$$

Here, FLOPs denote the total floating-point operations required for one forward propagation under a specified input resolution. Lower GFLOPs typically indicate faster inference and lower energy consumption, which is beneficial for real-time or low-compute scenarios.

### 3.3. Experimental Results

To validate both the segmentation performance and model complexity of the proposed method, DSMUNet was compared with a set of representative semantic-segmentation baselines on the constructed dataset. Specifically, TransUNet [25], DeepLabv3 [26], DeepLabv3+ [27], PSPNet [28], DCSAU-Net [29], ResUNet++ [30], U-Net [31], U-Net++ [32] and DSGNet [33] were selected for evaluation. The experimental results are shown in Table 3 and Table 4.

As shown in Table 3 and Table 4, DSMUNet demonstrates strong overall competitiveness on both the private dataset and the public Crack500 dataset, achieving a favorable balance among segmentation performance, model stability, and lightweight design.

From the results on the private dataset, DSMUNet achieves the best performance in terms of the Dice metric, indicating its strong ability to maintain overall overlap with crack regions. In terms of mIoU and F1, DeepLabv3 and TransUNet slightly outperform DSMUNet, but the performance gaps are relatively small. It should be noted that DeepLabv3 and TransUNet usually involve larger numbers of parameters and higher computational complexity. In contrast, DSMUNet achieves segmentation performance close to that of large-scale models while significantly reducing model size, demonstrating that the proposed model does not rely on large-scale parameter stacking, but instead achieves more efficient feature representation through lightweight structural design.

Compared with classical segmentation models, DSMUNet shows clear overall advantages on the private dataset. Compared with PSPNet, U-Net, and U-Net++, DSMUNet achieves better performance in Dice, mIoU, Recall, and F1, indicating that it can extract crack regions more completely and better balance false detections and missed detections under complex pavement backgrounds. Meanwhile, DSMUNet exhibits relatively small standard deviations overall, suggesting good stability across multiple independent experiments.

Compared with the lightweight models DCSAU-Net and DSGNet, DSMUNet also achieves better results on the private dataset. DSMUNet outperforms both DCSAU-Net and DSGNet in terms of Dice and mIoU, indicating that it can obtain better crack segmentation performance even with fewer parameters and lower computational cost. This further demonstrates that encoder-side spatial enhancement and decoder-side gated multi-scale fusion can effectively improve crack feature representation and structural continuity on the basis of a lightweight backbone.

On the public Crack500 dataset, DSMUNet also achieves favorable segmentation results. Specifically, DSMUNet obtains the highest Dice score among all compared models, indicating its strong advantage in crack-region overlap and overall segmentation completeness. Although DeepLabv3 slightly outperforms DSMUNet in mIoU, Recall, and F1, the performance gap is small, while DSMUNet has markedly lower model complexity. Compared with methods such as DCSAU-Net, U-Net, U-Net++, and PSPNet, DSMUNet maintains strong competitiveness in Dice, mIoU, and F1, indicating that the proposed model also has good generalization ability on the public dataset.

Overall, the results of the two sets of experiments show that the main advantage of DSMUNet does not lie in comprehensively surpassing all large-scale models on every single metric. Rather, it achieves segmentation performance comparable to, or even better than, that of several mainstream models while maintaining a lower number of parameters and reduced computational overhead. In particular, DSMUNet performs prominently on both the private dataset and the Crack500 dataset in terms of the Dice metric and stability across multiple experiments, suggesting that the model can effectively preserve the overall connectivity and segmentation consistency of crack regions.

In this study, five images were randomly selected from the test sets of the Crack500 dataset and the private dataset, respectively, for visual comparison. These samples include linear cracks, mesh cracks, and multi-morphology cracks, and some images contain various interference factors such as strong illumination and shadows. Therefore, they can effectively demonstrate the performance of different models under complex pavement environments.

**Figure 6:**
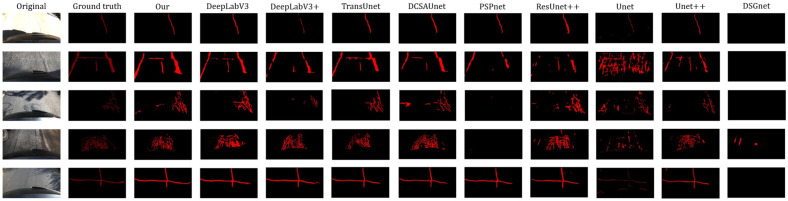
Visual comparison of predicted crack maps generated by different methods on the private dataset.

**Figure 7:**
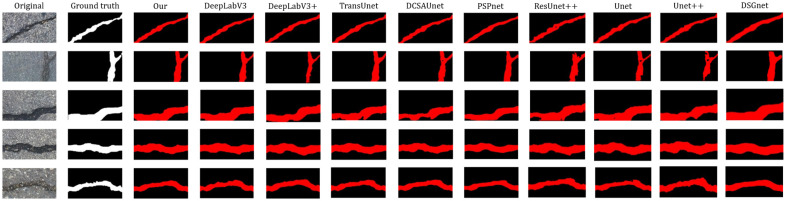
Visual comparison of predicted crack maps generated by different methods on the Crack500 dataset.

As shown in the segmentation comparisons in Figure 6 and Figure 7, TransUNet produces fewer false detections in the background and relatively clear crack boundaries. However, under certain environmental interferences, fine branches and detailed crack structures are easily missed. DeepLabv3 and DeepLabv3+ show good capability in preserving the overall connectivity of cracks and can integrate local crack regions effectively. Nevertheless, the edges of their segmentation masks are not sufficiently accurate, and missed detections and false detections still occur in regions with complex textures.

The predictions of PSPNet are relatively sparse, indicating insufficient feature extraction capability for fine cracks and mesh cracks, which easily leads to crack discontinuities and missed detections. DCSAU-Net achieves relatively stable segmentation of the main crack structures, but its boundary delineation becomes less clear under shadow and reflection interference. U-Net and U-Net++ produce relatively clean outputs, but they suffer from more frequent missed detections and false detections. ResUNet++ is prone to missed detections in images containing interference factors, and its boundary sharpness is limited.

In contrast, the proposed method can more stably preserve the overall continuity of cracks, produce clearer boundaries, and generate fewer false detections in shadowed and reflective regions. These results indicate that the proposed model has better robustness and segmentation performance under complex environmental conditions and diverse crack morphologies.

### 3.4. Ablation Study

To evaluate the contribution of each component module to the overall performance, ablation experiments were conducted in this study. Using the experimental dataset and identical hyperparameter settings, the following variants were respectively trained: single-module variants, namely DSC, SGE, and MFF; pairwise combinations, namely DSC+SGE, DSC+MFF, and SGE+MFF; and the full method integrating all improvements, denoted as Ours. Performance was evaluated using mIoU, Recall, F1, Dice, the number of parameters, and GFLOPs as metrics.

As shown in Table 5, after introducing DSC alone, the number of model parameters and computational cost are significantly reduced. However, segmentation metrics such as Dice, mIoU, Recall, and F1 all decrease, indicating that although depthwise separable convolution can effectively achieve lightweight modeling, simply replacing standard convolutions is insufficient to fully ensure fine-grained representation of crack regions.

After adding SGE on the basis of DSC, all metrics improve steadily, demonstrating that encoder-side spatial enhancement can strengthen crack-related features and suppress crack-like background interference, such as asphalt textures, shadow boundaries, and repair traces. In contrast, when only MFF is introduced, the corresponding metrics also improve to some extent, but the improvement is relatively limited. This suggests that multi-scale structural supplementation needs to be combined with stable crack feature representation from the front-end encoder to fully exert its effect.

The complete DSMUNet achieves the best overall performance in terms of Dice, mIoU, Recall, and F1, indicating that DSC, SGE, and MFF have a strong synergistic effect. Specifically, DSC ensures the lightweight nature of the model, SGE enhances the discriminative ability of crack features in the encoding stage, and MFF supplements multi-scale structural information in the decoding stage, thereby jointly improving the completeness and stability of crack segmentation.

For configurations without DSC, although some models can also achieve favorable segmentation results, their numbers of parameters and computational costs increase significantly. In comparison, DSMUNet achieves better segmentation performance while maintaining low model complexity, demonstrating that the proposed method achieves a better balance between segmentation performance and model complexity.

To further analyze the influence of loss-function weights on crack segmentation results, this study conducted comparative experiments on different combination ratios of cross-entropy loss and Dice loss. Since pavement crack pixels usually account for only a small proportion of the entire image, relying solely on cross-entropy loss may easily cause the optimization process to be dominated by the background class. In contrast, Dice loss can alleviate the foreground–background imbalance problem from the perspective of regional overlap. Therefore, this study adopts a combined loss formulation and evaluates different weight ratios.

As shown in Table 6, when the weight ratio of cross-entropy loss to Dice loss is 5:5, the model achieves the best overall performance, outperforming other ratio settings in terms of Dice, mIoU, Recall, and F1. Specifically, under the 5:5 combination, Dice reaches 78.10%, mIoU reaches 69.05%, and F1 reaches 77.74%, indicating that this ratio achieves a favorable balance between pixel-level classification constraints and regional-overlap constraints. In contrast, when the proportion of Dice loss is too high, the model places stronger emphasis on optimizing regional overlap, but the constraint on pixel-level boundary discrimination is relatively weakened. When the proportion of cross-entropy loss is increased, the model pays more attention to pixel classification, but its ability to comprehensively characterize fine cracks and imbalanced foreground regions may be affected.

Therefore, when selecting the loss-function ratio, Dice, mIoU, Recall, and F1 are used as the main criteria for evaluating the model’s segmentation capability for crack regions. Considering all these metrics, this study finally adopts $0.5L_{CE}+0.5L_{Dice}$ as the training loss function for subsequent comparative and ablation experiments.

## 4. Conclusions

To address the segmentation difficulty caused by diverse crack morphologies and interference from environmental factors such as shadows, repair marks, and background textures, as well as the fact that current existing methods find it difficult to maintain good segmentation accuracy under the premise of low computational cost, this study proposes a lightweight crack segmentation model, DSMUNet, based on a U-shaped encoder–decoder structure. By replacing standard convolution with depthwise separable convolution, the model scale and number of training parameters are reduced while maintaining feature representation capability. In addition, SGE spatial group enhancement is introduced at the encoder output, so as to suppress background noise, highlight crack features, and reduce the risk that interference features are amplified through skip connections, with only a small additional overhead. Finally, a multi-scale feature fusion module is proposed. By using multi-branch dilated depthwise convolution during the decoder feature fusion stage, structural information from different receptive fields is supplemented, and GroupNorm together with learnable gating factors is combined to reduce training fluctuation, thereby improving the overall connectivity of cracks and alleviating problems such as discontinuities and jagged edges. In addition, in order to verify the adaptability of the proposed method under complex environments, this study collected and constructed an asphalt pavement crack dataset containing interference factors such as strong light, shadows, occlusion, and repaired pavements, including a total of 3659 high-resolution images with pixel-level annotations completed.

The experimental results show that DSMUNet achieves favorable segmentation performance while maintaining a lightweight structure. The model contains only 0.55 M parameters and requires 21.708 GFLOPs, with an average inference latency of 5.42 ms. On the private dataset, DSMUNet achieves a Dice of 78.10%, an mIoU of 69.05%, a Recall of 75.81%, and an F1 score of 77.74%. On the Crack500 dataset, DSMUNet achieves a Dice of 87.12%, an mIoU of 79.57%, a Recall of 84.06%, and an F1 score of 87.40%. Compared with classical segmentation models such as PSPNet, DeepLabv3, and TransUNet, as well as lightweight models such as DCSAU-Net and DSGNet, DSMUNet achieves a favorable balance among segmentation performance, model size, and inference efficiency. These results indicate that the proposed method can effectively preserve the overall connectivity and segmentation stability of crack regions in complex pavement crack scenarios, providing a more efficient technical solution for road inspection and maintenance decision-making.

In future work, we will further conduct vehicle-mounted deployment validation in real-world road inspection scenarios. On the one hand, DSMUNet will be integrated into a vehicle-mounted image acquisition and edge computing platform to evaluate its real-time inference performance and segmentation stability under different vehicle speeds, illumination conditions, pavement materials, and complex weather conditions. On the other hand, the processing efficiency, localization consistency, and long-term operational reliability of the model in continuous video streams will be further assessed to verify its applicability in practical road maintenance and inspection tasks. In addition, future research will combine lightweight model compression, inference acceleration, and online updating strategies to further improve the deployment efficiency and environmental adaptability of DSMUNet on embedded vehicle-mounted devices.

## Figures and Tables

**Table 1:** Learned Final Values of *γ*.

Parameter	Value
Up stage 1	1.0254 ± 0.0697
Up stage 2	0.4652 ± 0.0972
Up stage 3	−0.1266 ± 0.0741
Up stage 4	0.8178 ± 0.0074

**Table 2:** Parameters for Dataset Construction.

Parameter	Value
Acquisition location	Changchun, Jilin Province, China
Acquisition platform	Vehicle-mounted camera platform
Vehicle speed	20–30 km/h
Original image resolution	2048 × 1080
Camera mounting position	Rear roof of the vehicle
Camera height above ground	2.3 m
Imaging angle	Vertically downward
Acquisition time	2023
Number of images	3659

**Table 3:** Results of Experiments on the Private Dataset.

Model	Params (M)	GFLOPs	Latency (ms)	Dice (%)	mIoU (%)	Recall (%)	F1 (%)
DSMUNet (our)	0.550	21.708	5.42	78.10 ± 0.13	69.05 ± 0.20	75.81 ± 0.29	77.74 ± 0.20
DeepLabv3	39.634	512.812	13.28	76.92 ± 0.08	69.15 ± 0.39	78.13 ± 0.86	77.86 ± 0.40
DeepLabv3+	39.757	145.819	9.24	74.88 ± 0.30	66.35 ± 0.36	69.27 ± 0.78	74.80 ± 0.40
PSPNet	27.708	85.035	8.28	72.36 ± 0.54	63.69 ± 0.16	66.83 ± 1.01	70.96 ± 0.80
DCSAU-Net	2.599	156.331	16.79	76.46 ± 0.33	68.10 ± 0.21	73.42 ± 0.74	76.74 ± 0.21
Res-UNet++	14.483	886.768	21.41	68.78 ± 1.34	63.01 ± 1.06	66.98 ± 3.58	69.50 ± 1.06
TransUNet	106.354	497.542	37.13	76.89 ± 1.08	69.41 ± 0.14	73.87 ± 0.66	78.14 ± 0.09
U-Net++	26.905	470.395	11.44	74.64 ± 1.16	65.48 ± 0.51	68.79 ± 1.11	73.83 ± 0.58
U-Net	17.263	501.693	10.64	74.75 ± 0.43	65.33 ± 0.37	68.69 ± 2.48	73.21 ± 0.94
DSGNet	0.488	6.164	8.71	53.91 ± 0.98	52.15 ± 0.77	52.86 ± 0.95	51.50 ± 0.67

**Table 4:** Experimental Results on the Crack500 Dataset.

Model	Params (M)	GFLOPs	Latency (ms)	Dice (%)	mIoU (%)	Recall (%)	F1 (%)
DSMUNet (our)	0.550	21.708	5.42	87.12 ± 0.10	79.57 ± 0.32	84.06 ± 3.19	87.40 ± 0.25
DeepLabv3	39.634	512.812	13.28	86.10 ± 0.11	79.90 ± 0.27	88.51 ± 0.38	87.65 ± 0.22
DeepLabv3+	39.757	145.819	9.24	83.21 ± 0.28	76.94 ± 0.15	83.62 ± 0.53	85.85 ± 0.73
PSPNet	27.708	85.035	8.28	84.72 ± 0.06	77.94 ± 0.15	85.26 ± 0.70	86.14 ± 0.12
DCSAU-Net	2.599	156.331	16.79	85.76 ± 0.12	78.77 ± 0.21	84.96 ± 0.74	87.13 ± 0.62
Res-UNet++	14.483	886.768	21.41	82.75 ± 0.46	76.31 ± 0.06	87.20 ± 0.87	84.85 ± 0.05
TransUNet	106.354	497.542	37.13	85.07 ± 1.05	78.54 ± 0.40	85.16 ± 1.73	86.60 ± 0.30
U-Net++	26.905	470.395	11.44	83.98 ± 0.20	76.92 ± 0.12	84.81 ± 0.62	85.32 ± 0.10
U-Net	17.263	501.693	10.64	83.24 ± 0.50	77.06 ± 0.24	83.45 ± 1.05	85.42 ± 0.20
DSGNet	0.488	6.164	8.71	79.15 ± 1.63	73.31 ± 1.66	82.83 ± 1.96	82.26 ± 1.47

**Table 5:** Results of Ablation Experiments.

Model	Params (M)	GFLOPs	Dice (%)	mIoU (%)	Recall (%)	F1 (%)
Ours	0.550	21.708	78.10 ± 0.13	69.05 ± 0.20	75.81 ± 0.29	77.74 ± 0.20
Baseline+SGE	17.263	501.893	75.89 ± 1.44	67.32 ± 0.45	71.08 ± 1.37	75.88 ± 0.60
Baseline+SGE+MFF	17.336	509.757	76.45 ± 1.10	67.75 ± 0.46	72.67 ± 1.00	76.36 ± 0.49
Baseline+MFF	17.336	509.557	75.56 ± 1.02	67.68 ± 0.43	71.94 ± 1.86	76.28 ± 0.48
Baseline+DSC	0.513	17.676	74.10 ± 1.23	64.15 ± 0.89	67.70 ± 1.84	71.53 ± 1.21
Baseline+DSC+SGE	0.513	17.776	76.41 ± 0.66	67.50 ± 0.24	72.01 ± 1.02	76.09 ± 0.26
Baseline+DSC+MFF	0.550	21.608	74.97 ± 0.86	65.52 ± 0.60	69.91 ± 1.39	73.83 ± 1.07

**Table 6:** Performance Comparison under Different Loss Function Settings.

Loss Ratio	Dice (%)	mIoU (%)	Recall (%)	F1 (%)
$0.2L_{CE}+0.8L_{Dice}$	77.46 ± 0.78	68.91 ± 0.33	75.46 ± 1.30	77.59 ± 0.40
$0.3L_{CE}+0.7L_{Dice}$	76.95 ± 0.73	68.45 ± 0.66	73.85 ± 1.19	77.10 ± 0.71
$0.4L_{CE}+0.6L_{Dice}$	77.65 ± 0.36	68.44 ± 0.56	74.59 ± 2.65	77.10 ± 0.60
$0.5L_{CE}+0.5L_{Dice}$	78.10 ± 0.13	69.05 ± 0.20	75.81 ± 0.29	77.74 ± 0.20
$0.6L_{CE}+0.4L_{Dice}$	77.34 ± 0.66	68.50 ± 0.47	75.26 ± 1.82	77.17 ± 0.55

## Data Availability

The original contributions presented in the study are included in the article; further inquiries can be directed to the corresponding author.
